# Comparison between toric and spherical phakic intraocular lenses combined with astigmatic keratotomy for high myopic astigmatism

**DOI:** 10.1186/s40662-017-0085-7

**Published:** 2017-08-18

**Authors:** Lin-Yan Zheng, Shuang-Qian Zhu, Yan-Feng Su, Hu-Yong Zou, Qin-Mei Wang, A-Yong Yu

**Affiliations:** The Eye Hospital of Wenzhou Medical University, 270 Xueyuan West Road, Wenzhou, 325000 Zhejiang People’s Republic of China

**Keywords:** Astigmatism, High myopia, Phakic intraocular lenses, Astigmatic keratotomy

## Abstract

**Background:**

To compare the outcomes of a toric phakic intraocular lens (PIOL) and a spherical PIOL combined with astigmatic keratotomy (AK) for the correction of high myopic astigmatism.

**Methods:**

This study enrolled patients with high myopic astigmatism, including 30 eyes (22 patients) that received a toric PIOL implantation (TICL group), and 32 eyes (24 patients) that received combined AK and a spherical PIOL implantation (AK+ ICL group). The outcomes were compared between the two groups before surgery, and at the following time points after surgery: 1 week, 1, 3, 6 months, and 1, 2 years.

**Results:**

Preoperatively, the mean manifest spherical equivalent (SE) was −14.14 ± 2.12 D in the TICL group and −14.83 ± 2.79 D in the AK + ICL group (*P* = 0.28), and the mean manifest refractive cylinder, −2.87 ± 1.09 D and −2.58 ± 0.85 D, respectively (*P* = 0.28). Two years postoperatively, the mean safety index was 1.53 ± 0.55 in the TICL group and 1.60 ± 0.70 in the AK + ICL group (*P* = 1.00), and the mean efficacy index, 1.18 ± 0.45 and 1.38 ± 0.52, respectively (*P* = 0.86). The mean manifest refractive cylinder correction was 1.94 ± 1.07 D in the TICL group and 1.39 ± 0.71 D in the AK + ICL group (*P* = 0.02). The mean changes in SE and refractive cylinder from 1 week to 2 years were less than 0.50 D in both groups.

**Conclusions:**

Both TICL implantation and AK + ICL implantation are a good alternative for correction of astigmatism in addition to high myopia. TICL implantation has better predictability in correction of high myopic astigmatism.

**Trial registration:**

NCT03202485

## Background

Phakic intraocular lenses (PIOLs) are regarded as an alternative to the current modalities of refractive correction for high myopia. The Implantable Collamer Lens (ICL; STAAR Surgical, Switzerland) is one of the options of regularly used PIOLs, and demonstrates its safety and effectiveness in correcting spherical refractive errors. Considering that astigmatism is common in highly myopic eyes [1], a toric ICL (TICL) implantation or combined astigmatic keratotomy (AK) and ICL (AK + ICL) implantation may be a feasible approach to correct both spherical and cylindrical errors in these cases, offering optimum vision without the use of spectacles for correcting astigmatism. Many studies have compared outcomes between the TICL and excimer laser corneal refractive surgery [2–4], which demonstrated clear superiority of the TICL for high myopic astigmatism. However, to our knowledge, no comparisons between the TICL and the AK + ICL implantation have been published. This prospective study was to compare the postoperative outcomes of TICL and AK + ICL implantation for the correction of high myopia with astigmatism.

## Methods

### Patients

This prospective comparative study enrolled 30 eyes of 22 patients having TICL implantation, and 32 eyes of 24 patients having AK + ICL implantation for the correction of high myopic astigmatism. All patients underwent a complete ophthalmologic examination including slit-lamp biomicroscopy, corneal topography, corneal pachymetry, and dilated fundoscopy. The inclusion criteria included: 1) age ranged from 18 to 40 years, 2) myopia greater than −8.00 diopters (D), and refractive cylinder in the range of 1.50 D to 5.50 D, 3) for the patients who were planned to undergo AK + ICL implantation, the axial difference between the corneal astigmatism and the manifest refractive astigmatism was less than 10 degrees, 4) a stable refractive error during the previous 2 years, 5) anterior chamber depth more than 2.8 mm, 6) endothelial cell density (ECD) more than 2500 cells/mm^2^, and 7) scotopic pupillary diameter less than 7 mm. None of the subjects had significant irregular astigmatism, corneal pathological changes, glaucoma, ocular inflammation, or previous ocular trauma or surgery.

This study followed the tenets of the Declaration of Helsinki. All subjects provided informed consent and approval was obtained from the Institutional Review Board of the Eye Hospital of Wenzhou Medical University.

### Follow-up examinations

Postoperative follow-up visits were at 1 day, 1 week, 1, 3, and 6 months, and 1, 2 years. Uncorrected visual acuity (UCVA), best corrected visual acuity (BCVA), refraction, slit-lamp biomicroscopy, ECD, intraocular pressure, and fundus examination were performed.

### Surgical procedure

The PIOL size and power were determined following the manufacturer’s recommendations. The PIOL implantation technique for the TICL group was as follows: two peripheral iridectomies were made with a neodymium: YAG laser preoperatively. On the day of surgery, the zero horizontal meridian was marked using a slit-lamp while the patient was sitting upright. Surgery was performed under pupil dilation and topical anesthesia. A Mendez ring was used for measuring the required rotation from the horizontal meridian. The TICL was inserted through a 3.0 mm clear corneal incision with viscoelastics into the anterior chamber. Then, the TICL was placed in the posterior chamber, and was exactly aligned to the cylinder axis of the patient’s required cylinder correction. The remaining viscoelastics were completely irrigated out of the anterior chamber with a balanced salt solution. No acetylcholine chloride was administrated for miosis. The incision was closed by hydration without sutures.

For the AK + ICL group, the procedure was the same as mentioned above except that AK was performed before ICL implantation and no rotation of ICL was required. The procedure of AK was briefly introduced as follows: after marking the proposed AK incision site on the corneal epithelium with a marker, the paired arcuate corneal incisions perpendicular to the meridian with stronger manifest refractive power were made according to the Lindstrom nomogram by a diamond knife adjusted to the planned incision depth. The incisions were 95% of the peripheral corneal thickness with an optical zone diameter of 7 mm. Finally, the incisions were irrigated with a balanced salt solution. The manifest refractive astigmatism was selected as the target correction.

The surgical decision of TICL or AK in an eye with astigmatism was based on the surgeon’s discretion and the patient’s ability to afford the surgery. Where both options were considered appropriate, we explained both options to the patients and they were asked to make a choice.

### Postoperative care

The patient was given eye drops of 0.5% levofloxacin (Cravit; Santen Pharmaceutical Co. Ltd., Japan) and 0.1% fluorometholone (Flumetholon; Santen Pharmaceutical Co. Ltd., Japan), to be used four times a day for the first postoperative week and three times a day in the second postoperative week; the eye drops were to be discontinued from the third week onwards. The topical NSAID (Pranopulin; Santen Pharmaceutical Co. Ltd., Japan) was used four times a day for the first postoperative month. Patients were also given vitamin A palmitate eye gel (Oculotect, Novartis Ophthalmics AG, Switzerland) for use four times a day for maintenance of tear film and a regular ocular surface.

### Statistical analysis

Data were collected on standardized case-report forms, and then entered into a central database for analysis. Statistical analysis was performed with commercial software (SPSS, ver. 19.0; SPSS, Chicago, USA). Normality of data was checked using the Kolmogorov-Smirnov test. Descriptive statistics for continuous variables were calculated as means and standard deviations (SDs). For averaging, visual acuity was converted to logMAR value, and was back-calculated to Snellen acuity where noted. Astigmatism was analyzed by the vector analysis [5]. Wilcoxon test and one-way ANOVA was used. The level of significance was set at *P* < 0.05.

## Results

Table 1 shows the patients’ demographics. Before surgery, there were no significant differences between the 2 groups in age, sex, manifest spherical equivalent (SE), manifest cylinder, UCVA, or BCVA.

**Table 1:** Preoperative patient demographics

Characteristic	TICL Group	AK + ICL Group	*P* Value
Age(years)			
Mean ± SD	25.38 ± 3.89	24.29 ± 6.20	0.405
Range	19 to 35	18 to 38	
Sex (% female)	56.7%	56.3%	0.129
Manifest spherical equivalent (D)			
Mean ± SD	−14.14 ± 2.12	−14.83 ± 2.79	0.278
Range	−10.00 to −17.50	−9.50 to −19.50	
Manifest cylinder (D)			
Mean ± SD	−2.87 ± 1.09	−2.58 ± 0.85	0.288
Range	−1.50 to −5.50	−1.50 to −5.50	
Best corrected visual acuity (LogMAR)			
Mean ± SD	0.25 ± 0.21	0.24 ± 0.35	0.887
Range	0.60 to 0.00	1.00 to −0.18	
Uncorrected visual acuity (LogMAR)			
Mean ± SD	1.05 ± 0.05	1.10 ± 0.06	0.383
Range	2.00 to 0.70	2.00 to 0.60	

*TICL* toric implantable collamer lens, *AK* astigmatic keratotomy, *ICL* implantable collamer lens

### Safety

**Fig. 1:**
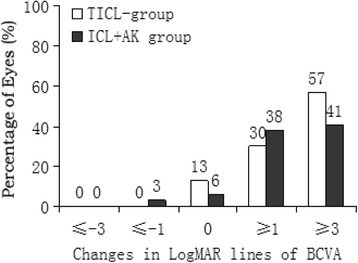
Distribution of best corrected visual acuity (BCVA) in the TICL and AK + ICL groups 2 years after surgery

At all postoperative periods, there was no between-group difference in the BCVA or the safety index (mean postoperative BCVA/mean preoperative BCVA) (Table 2), and BCVA was improved significantly from the baseline in both groups (*P* < 0.001). Figure 1 shows the percentage of eyes with no change in BCVA, gained 1 or more lines of BCVA, and lost 1 line of BCVA 2 years after surgery.

**Table 2:** Comparison of postoperative outcomes between TICL and AK + ICL groups

Variable	TICL Group	AK + ICL Group	*P* value
Manifest spherical equivalent (D)			
1 week	−0.27 ± 0.79	−0.12 ± 0.65	0.433
1 month	−0.28 ± 0.95	−0.42 ± 0.89	0.648
3 months	−0.30 ± 0.81	−0.54 ± 0.66	0.571
6 months	−0.17 ± 0.82	−0.32 ± 0.89	0.913
1 year	−0.56 ± 0.65	−0.32 ± 1.01	0.517
2 years	−0.46 ± 0.64	−0.44 ± 0.91	0.910
Manifest cylinder (D)			
1 week	−0.88 ± 0.80	−1.04 ± 0.60	0.124
1 month	−0.94 ± 0.47	−1.38 ± 0.72	0.045
3 months	−0.98 ± 0.54	−1.39 ± 0.57	0.043
6 months	−0.89 ± 0.99	−1.28 ± 0.63	0.038
1 year	−0.92 ± 0.98	−1.22 ± 0.64	0.043
2 years	−0.93 ± 0.78	−1.19 ± 0.65	0.024
Best corrected visual acuity (LogMAR)			
1 week	0.14 ± 0.23	0.15 ± 0.35	0.971
1 month	0.13 ± 0.24	0.09 ± 0.25	0.450
3 months	0.12 ± 0.26	0.10 ± 0.32	1.000
6 months	0.13 ± 0.27	0.08 ± 0.25	0.362
1 year	0.11 ± 0.23	0.11 ± 0.26	0.818
2 years	0.09 ± 0.26	0.10 ± 0.36	0.891
Uncorrected visual acuity (LogMAR)			
1 week	0.29 ± 0.24	0.32 ± 0.27	0.701
1 month	0.28 ± 0.20	0.30 ± 0.32	0.611
3 months	0.29 ± 0.19	0.31 ± 0.15	0.794
6 months	0.28 ± 0.17	0.29 ± 0.23	0.491
1 year	0.25 ± 0.20	0.26 ± 0.32	0.775
2 years	0.21 ± 0.23	0.20 ± 0.33	0.966
Safety index			
1 week	1.46 ± 0.38	1.31 ± 0.51	0.102
1 month	1.48 ± 0.45	1.48 ± 0.40	0.895
3 months	1.59 ± 0.86	1.62 ± 0.53	0.581
6 months	1.60 ± 0.80	1.82 ± 0.82	0.856
1 year	1.68 ± 0.89	1.70 ± 0.78	0.612
2 years	1.53 ± 0.55	1.60 ± 0.70	1.000
Efficacy index			
1 week	1.04 ± 0.35	0.94 ± 0.24	0.728
1 month	1.13 ± 0.40	0.86 ± 0.13	0.161
3 months	1.12 ± 0.59	1.14 ± 0.28	0.259
6 months	1.14 ± 0.53	1.23 ± 0.29	0.354
1 year	1.17 ± 0.58	1.17 ± 0.42	0.748
2 years	1.18 ± 0.45	1.38 ± 0.52	0.816

*TICL* toric implantable collamer lens, *AK* astigmatic keratotomy, *ICL* implantable collamer lens

### Efficacy

**Fig. 2:**
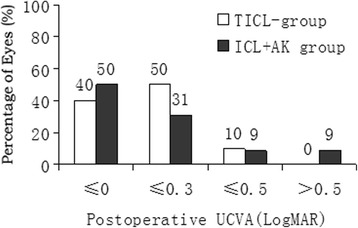
Proportion of eyes with uncorrected visual acuity (UCVA) in LogMAR in the TICL and AK + ICL groups 2 years after surgery

At all postoperative periods, there was no between-group difference in the UCVA or the efficacy index (mean postoperative UCVA/mean preoperative BCVA) (Table 2), and UCVA was improved significantly from the baseline in both groups (*P* < 0.001). Figure 2 shows the percentage of eyes that had a LogMAR UCVA of 0 or better, and 0.5 or worse 2 years after surgery.

### Predictability

#### Spherical equivalent

**Fig. 3:**
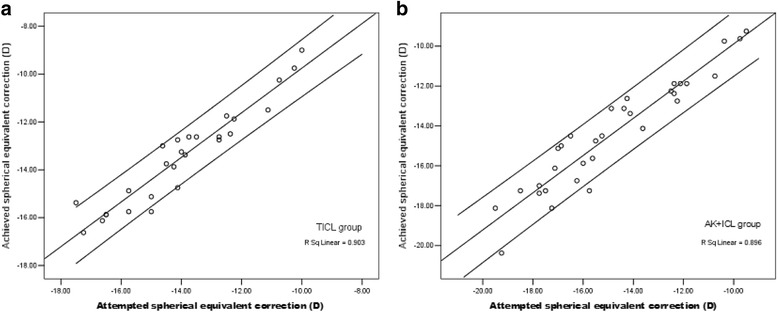
Scattergrams illustrating the spherical equivalent attempted versus achieved 2 years after surgery. **a** TICL group; **b** AK + ICL group

**Fig. 4:**
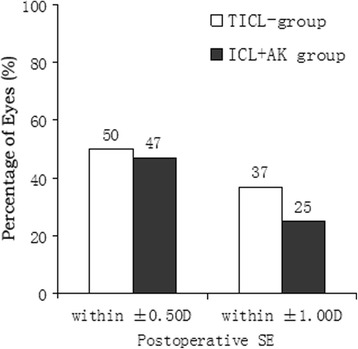
Percentage of eyes within ±0.50 D and ±1.00 D of the attempted correction of spherical equivalent (SE) in the TICL and AK + ICL groups

At all postoperative periods, SE was significantly improved from the baseline in both groups (*P* < 0.001), and had no significant between-group difference (Table 2). Figure 3 shows a scatterplot of the attempted versus the achieved correction of SE 2 years after surgery. Figure 4 shows the percentage of eyes within ±0.50 D and within ±1.00 D of the attempted correction of SE 2 years after surgery.

#### Astigmatism

**Fig. 5:**
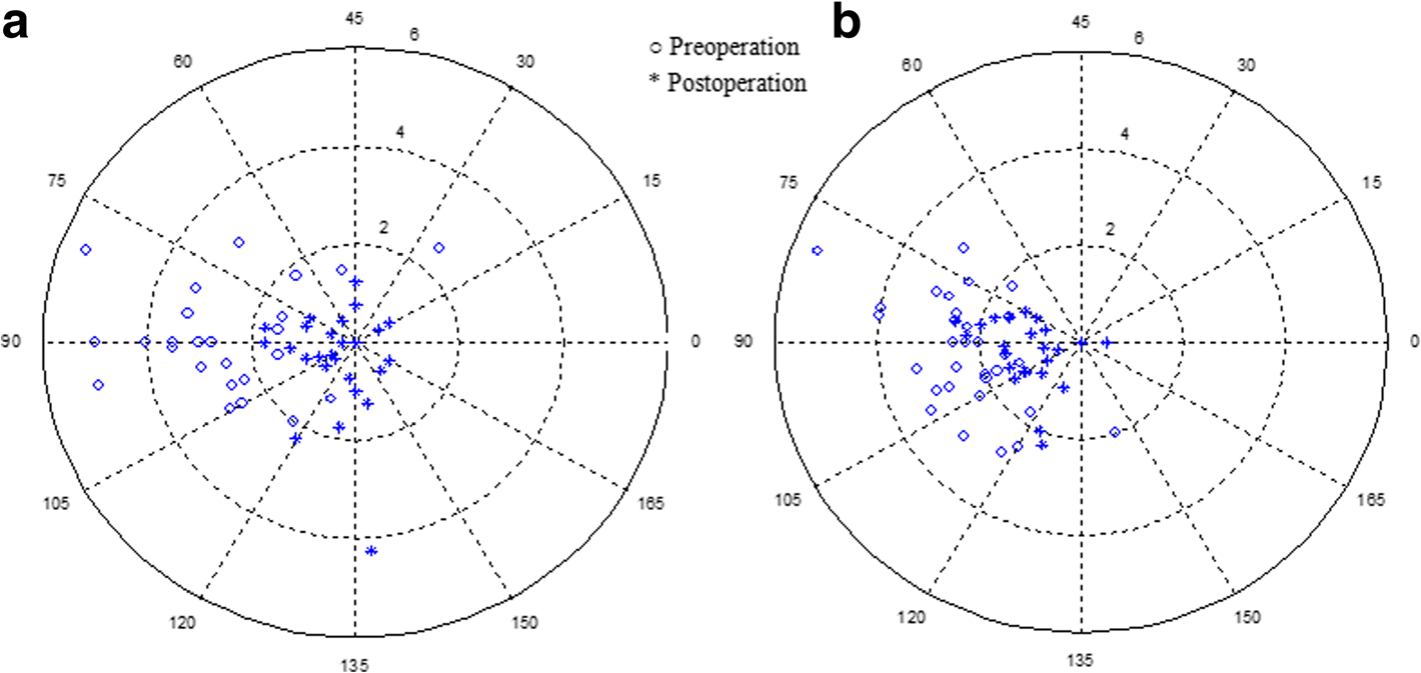
Double-angled polar plot of astigmatism before and 2 years after surgery. **a** TICL group; **b** AK + ICL group. The dots closer to the origin indicates less astigmatism

At all postoperative periods, the manifest astigmatism was significantly improved from the baseline in both groups (*P* < 0.001), and had a significant between-group difference except for 1 week (Table 2). Figure 5 shows the manifest astigmatism that was improved significantly in both groups 2 years postoperatively.

### Stability

#### Spherical equivalent

**Fig. 6:**
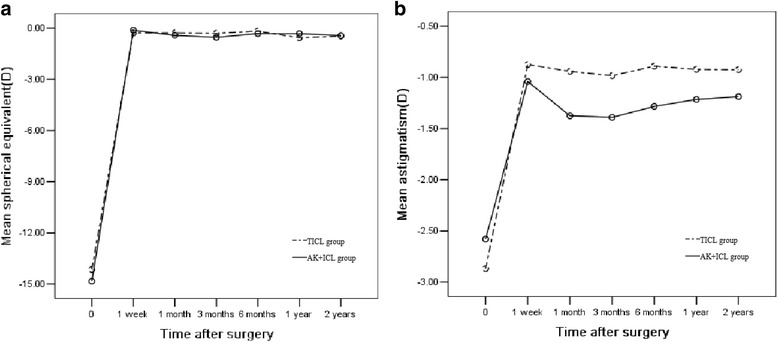
Time course of manifest spherical equivalent (**a**) and manifest cylinder (**b**) in the TICL and AK + ICL groups

Figure 6 shows the change in the manifest SE. There was no significant difference between each postoperative time in both groups. The mean change in manifest SE from 1 week to 2 years was 0.19 ± 0.32 D in the TICL group and 0.32 ± 0.40 D in the AK + ICL group (*P* = 0.67).

#### Astigmatism

Figure 6 shows the change in the manifest cylinder. There was no significant difference between each postoperative time in both groups. The mean change in manifest cylinder from 1 week to 2 years was 0.05 ± 0.23 D in the TICL group and 0.15 ± 0.18 D in the AK + ICL group (*P* = 0.30).

There were no significant differences in BCVA, UCVA, safety index or efficacy index between each postoperative time for both groups.

### Complications

All surgeries were uneventful and there were no significant intraoperative complications. One week postoperatively, 1 eye in the TICL group and 2 eyes in the AK + ICL group experienced glare at night, which disappeared after 3 months. There were no clinically significant axis deviation, pupillary block, cataract formation, or pigment dispersion syndrome during the 2-year observation period. There were no noted complications of AK such as corneal perforation, delayed corneal epithelialization, corneal edema, or infection.

## Discussion

Astigmatism reduces visual quality by causing glare, monocular diplopia, asthenopia, and vision distortion after the spherical refractive error is corrected by a spherical PIOL in high myopic astigmatism patients. The three primary treatment modalities to reduce pre-existing astigmatism at the time of PIOL surgery are AK, limbal relax incisions (LRIs), and the implantation of toric PIOLs. AK has succeeded in correcting astigmatism and been shown to be a simple and effective procedure [6, 7]. Compared with LRIs, AK has the advantage of more effectively reducing astigmatism with shorter incision lengths due to closer proximity to the visual axis. Toric PIOL implantation, as a correction of high myopic astigmatism, is safe, effective, and shows good predictability and stability. Limitations of toric PIOLs include the potential IOL rotation off alignment postoperatively and cost of the procedure. To our knowledge, no comparative study between TICL implantation and AK + ICL implantation has been performed.

The current study shows that the refractive outcomes and improvement in UCVA and BCVA were rapidly achieved and remained stable in the TICL group throughout the follow-up period. This agrees with the reports in the literature [8–10]. The United States FDA clinical trial of TICL [11] demonstrated that astigmatism was reduced from 1.93 ± 0.84 D to 0.51 ± 0.48 D, and postoperative BCVA was improved by at least one line in 76.4% of patients, postoperative UCVA was better than or equal to preoperative BCVA in 76.5% of patients. In this study, TICL group showed that astigmatism was reduced from −2.87 ± 1.09 D to −0.93 ± 0.78 D, and postoperative BCVA was improved at least one line in 87% of patients while postoperative UCVA was better than or equal to preoperative BCVA in 67% of patients. The difference in postoperative cylinder may be due to the different baseline cylinder. The baseline cylinder is higher in our study than in Sanders’s study (2.87 D vs. 1.93 D) [11]. The results of this study are comparable to the previous studies on TICL correcting high myopic astigmatism, indicating that the refractive outcomes of TICL in our study are common to the procedure.

However, the postoperative clinical outcomes of AK + ICL implantation in high myopic astigmatism patients have not been fully elucidated. In this study, the AK + ICL group showed that astigmatism was reduced from −2.58 ± 0.85 D to −1.19 ± 0.65 D. It was reported that corneal astigmatism was reduced from 2.90 ± 0.78 D to 0.89 ± 0.52 D in patients having AK alone, and from 2.97 ± 1.01 D to 1.02 ± 0.45 D in those having AK combined with cataract surgery [6]. In the AK + ICL group, 72% of cases were within ±1.00 D of the attempted SE correction. The power calculation of ICL is not affected by AK because AK does not change SE, and the paired arcuate incisions of AK achieved a more ideal corneal sphericity than that preoperatively, which is known as the coupling effect [12]. In the AK + ICL group, the postoperative BCVA was improved by at least one line in 79% of patients, the postoperative UCVA was better than or equal to preoperative BCVA in 66% of patients. As far as stability, both groups seem to have similar variations of SE and astigmatism during the period of follow-up, which was less than 0.50D on average. These results demonstrated AK + ICL group and TICL group have comparable safety and efficacy in correction of high myopic astigmatism.

But the magnitude of astigmatism correction in the TICL group was significantly greater than that in the AK + ICL group (1.94 ± 1.07 D vs. 1.39 ± 0.71 D), indicating that TICL implantation had better predictability in correcting high myopic astigmatism compared to AK + ICL implantation. Generally, target of cylinder correction in TICL is nearly zero. However, most of the AK nomogram is not designed to target 100% of preoperative cylinder correction to reduce the risk of overcorrection. The magnitude of astigmatism correction of AK depends on the size of the central optical zone and the number, depth, and length of incisions. In this study, the size of the optical zone and the number of incisions were kept constant, and the length of incisions was determined according to the same nomogram. The shallower cut may be involved although the diamond knife was adjusted to 95% of the peripheral corneal thickness. The lack of predictability of astigmatism correction by AK is probably related to the variation of achieved depth of the incision [13]. In addition, the smaller change in astigmatism produced after AK may be due to intraoperative axis misalignment. Although clinically significant axis deviation was not found in this study, intraoperative misalignment could not be ruled out as a factor that induced the postoperative axis deviation, which influences the efficacy of AK. With the advent of femtosecond laser technology, instead of using diamond knife, the femtosecond laser assisted AK may reduce the deviation of depth and size of corneal incisions to a minimum because all the parameters (length, depth, and site) are controlled and executed with submicron accuracy.

In this study, glare was noted in a total of 3 eyes, including 1 eye in the TICL group and 2 eyes in the AK + ICL group, which disappeared 3 months after surgery. The presence of glare after surgery may be associated with a small optical zone, large pupil and increased irregular astigmatism. In this study, the optical zone of PIOL and pupil size in both groups were comparable, and the optical zone of AK was set at 7 mm to eliminate induced irregular astigmatism. However, considering that AK might have a slower wound-healing process, and more time is possibly needed to restore stable corneal architecture, the patients in the AK + ICL group might experience more glare than those in the TICL group. Chang et al [14] reported that 1 eye suffered from persistent halo and disappeared 1 year after TICL implantation. No patients reported glare 1 year after AK in Akura’s study [6]. Budak et al [15] reported 1 patient that complained of glare for 1 month after LRI. Patients might have a certain degree of adaptation to glare. In addition, no anterior subcapsular opacification was observed in both groups during the follow-up period. An incidence rate of postoperative anterior subcapsular opacification had been reported from 2.7% to 7% in previous studies of ICL [10, 16, 17]. Lindland and co-authors [18] found that anterior subcapsular opacification may result from the contact between the PIOL and crystalline lens. The difference in occurrence of anterior subcapsular opacification may be related to different sample sizes, follow-up periods, and surgical techniques employed.

## Conclusions

In conclusion, our results demonstrated that both TICL implantation and AK + ICL implantation are good alternatives for the correction of astigmatism as well as high myopia. Compared with AK + ICL implantation, TICL implantation has better predictability in correcting high myopic astigmatism and preserves corneal contour by avoiding additional corneal incisions due to AK. AK + ICL implantation has advantages of lower cost to the patient and avoids the potential rotation off alignment due to TICL. The limitations of this study were a relatively small sample size and lack of randomization. Further investigations with a larger number of subjects and randomization are warranted.
